# Need to address the gender disparities in neurosurgery in India

**DOI:** 10.1097/MS9.0000000000001544

**Published:** 2023-12-04

**Authors:** Ayush Anand, Ismail A. Ibrahim, Priyangi Kathayat, Ayesha Ansari, Yash Aggarwal, Riddhpreet Kaur Wahi, Prakasini Satapathy, Sarvesh Rustagi

**Affiliations:** a B.P. Koirala Institute of Health Sciences, Dharan, Nepal; b Fenerbahçe Üniversitesi, Faculty of Health Sciences, Physiotherapy and Rehabilitation Pr, Istanbul, Turkey; c Smt NHL Municipal Medical College, Ahmedabad, Gujarat; d Jawaharlal Nehru Medical College, Datta Meghe Institute of Higher Education and Research, Wardha; e Global Consortium of Medical Education and Research, Pune; f Grant Government Medical College and Sir J.J Group of Hospitals, Mumbai, Maharashtra; g Government Institute of Medical Sciences, Greater Noida; h Center for Global Health Research, Saveetha Medical College and Hospital, Saveetha Institute of Medical and Technical Sciences, Saveetha University, Chennai; i School of Applied and Life Sciences, Uttaranchal University; j School of Pharmacy, Graphic Era Hill University, Dehradun, Uttarakhand, India

*Dear Editor*,

Despite the rise in female medical students worldwide, they continue to be under-represented in surgical specialties, especially orthopedic surgery, thoracic surgery, and neurosurgery^1–4^. Explicitly focusing on neurosurgery, this field has been historically dominated by men, and despite the increasing number of female neurosurgeons, it will take decades to counter this gender disparity^4–9^. Even in developed countries like the USA and Canada, only 12% of neurosurgeons are female^4^. Similarly, across Europe, ~12% of neurosurgeons are female^7^. In India, the condition is even worse, with females contributing only 2.5% of neurosurgeons^4^. This consistently low representation of women in neurosurgery causes a significant deprivation of female role models available to mentor the residents and junior doctors^10^. However, the issue of gender disparity in the field of neurosurgery worldwide is often misunderstood and misconstrued. Sometimes, this phrase is used for ulterior motives. The prevailing belief is that women are overlooked in neurosurgery selection programs, but the actual situation is more complex. Understanding the current situation globally and regionally is critical to address this issue.

Many women hesitate to pursue a career in neurosurgery for various personal reasons, which are often at the forefront of their decision-making. Neurosurgery is an arduous profession, and even for men, it can be challenging to endure. Consequently, women are frequently discouraged from entering this specialty. Studies across the globe have revealed the various barriers (Fig. 1) that impede women’s entry into neurosurgery, notably in academic settings, including the lack of role models, gender discrimination concerns, and work-life balance challenges^3,4,8,11–14,15,16^. A survey conducted amongst medical students in India revealed that the most common (87.4%) barrier to pursuing neurosurgical residency was lacking prospects and inadequate mentorship, whereas 65.2% of students voted it as an absence of a female role model^15^. Another study showed that approximately one-third of neurosurgeons in India are doing residency and are younger than 40^16^. Notably, three-fourths of female neurosurgeons were discouraged from joining neurosurgery residency^16^. Though most women neurosurgeons received good support from the department, female peers, and their families, approximately two-fifths reported having faced gender discrimination in the workplace and believed that women-only forums could help address various issues arising from the gender gap^16^. Also, most female neurosurgeons had difficulty maintaining work-life balance^16^. Similarly, Pahwa *et al.* ^15^ also reported problems in maintaining work-life balance. In addition, female medical students perceived maternity and neurosurgery as incompatible^15^. All these reveal a need for holistic assessment and development of mentorship initiatives to counter the gender disparities in neurosurgery (Fig. 2)^17^.

**Figure 1:**
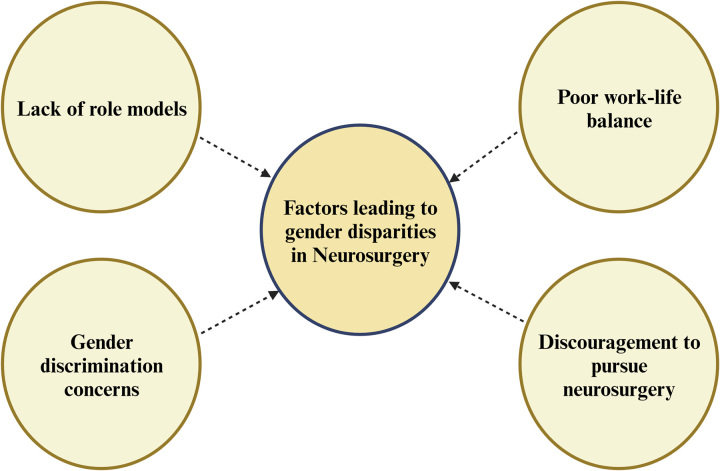
Factors leading to gender disparities in neurosurgery [Created with BioRender.com].

**Figure 2:**
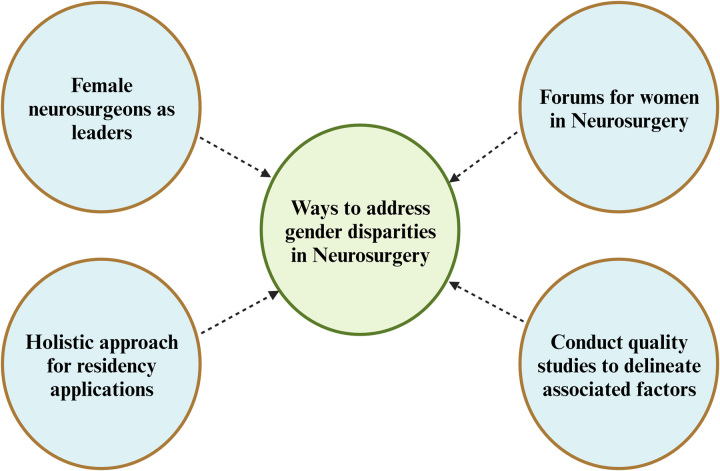
Ways to address gender disparities in neurosurgery [Created with BioRender.com].

Work is being done by projecting female surgeons as leaders and heads of departments across some prominent institutes in India, aiming to act as role models and motivate female medical students to pursue neurosurgery and promote a better workplace environment for female neurosurgeons^18^. Under the aegis of the Neurological Society of India, the inauguration of Women in Neurosurgery by Dr T. S. Sanaka (the first female neurosurgeon in Asia and India) is one of the forums to provide a platform to promote women as leaders in neurosurgery and address the issues arising due to gender barriers^18^. In India, the selection system for neurosurgery residency is strictly academic, based on the scores attained in the entrance exam. This may not be sufficient to ensure equity in neurosurgery in India. There is a need to adapt the system to be more holistic and consider individual qualities/traits and life-experiences in the selection process for residents. Though conveniently suggested, this may be difficult to address in Indian academia, as it will involve some significant structuring of the selection process.

While we acknowledge the confinements in gender variables, numerous studies have concluded that biodiversity elevates overall performance and augments the productivity and stability of the system^19^. The concern that this emphasis on inclusivity would undermine the field’s quality standards is one prevalent objection to the diversification of the neurosurgical workforce. However, diversification has been empirically shown to improve performance without compromising the integrity of systems and bring promising innovations to the table in various other professional situations^20,21^. This will help provide quality healthcare services with improved efficacy, which is particularly important in developing countries like India, where resources are limited^22^. Therefore, addressing the gender gap is a reasonable approach to expect comparable successful outcomes in neurosurgery.

In addition to the clinical workforce, it is essential to address the gender disparity in research and authorship positions in Neurosurgery, as they help in career progression. Though there has been an increase in women’s participation in academic research, only 3.6% had females as senior authors in developed countries like the USA and Canada^23^. Also, only 8.3% of female neurosurgeons have first-author abstract presentations, which is extremely low^24^. These abstract conferences are a great way to get exposure to academic research, and for building connections for career advancement. Studies have revealed that while the overall number of women in neurosurgery remains relatively constant from residency to academic appointments, there is a significant drop in the number of women holding full professorships compared to those in assistant and associate professor positions^25,26^. This decrease may be due to limited opportunities secondary to lesser academic research participation. A recent study has suggested addressing the disparity in academic research productivity by increasing the number of female editors^27^. Focusing on India, the data regarding the involvement of Indian female neurosurgeons in academic research is limited. Hence, more studies are required to delineate the associated factors further and address these issues to facilitate a smooth career progression.

Despite the available evidence, there are certain aspects which need further inquiry. The studies done in India so far lack detailed analysis and appropriate study design to identify factors influencing gender disparity in India. Also, the participation and leadership of women neurosurgeons in research is an area that needs to be explored further in Indian academia. The key is to conduct longitudinal studies with appropriate randomization with qualitative studies focusing on various aspects of gender disparity leading to problems in the workplace. These studies will help identify associated factors and devise effective intervention strategies to address the gender gap in neurosurgery in India.

In addition to the clinical workforce, it is essential to address the gender disparity in research and authorship positions in Neurosurgery, as they help in career progression. Though there has been an increase in women’s participation in academic research, only 3.6% had females as senior authors in developed countries like the USA and Canada^23^. Also, only 8.3% of female neurosurgeons have first-author abstract presentations, which is extremely low^23^. These abstract conferences are a great way to get exposure to academic research, and for building connections for career advancement. Studies have revealed that while the overall number of women in neurosurgery remains relatively constant from residency to academic appointments, there is a significant drop in the number of women holding full professorships compared to those in assistant and associate professor positions^25,26^. This decrease may be due to limited opportunities secondary to lesser academic research participation. Addressing this aspect is critical in the long run, as female neurosurgeons are more likely to complete their fellowships than their male counterparts, which can help develop a better-trained workforce^28^. A recent study has suggested addressing the disparity in academic research productivity by increasing the number of female editors^27^. Focusing on India, the data regarding the involvement of Indian female neurosurgeons in academic research is limited. Hence, more studies are required to delineate the associated factors further and address these issues to facilitate a smooth career progression.

Neurosurgery is a demanding field requiring round-the-clock vigilance as it deals with emergency surgeries and critical medical cases. The irregular and long working hours can be challenging for many individuals, including women. Therefore, it is essential to encourage women to explore careers in neurosurgery while being mindful of the unique demands it presents. Support and cooperation from colleagues can play a pivotal role in facilitating gender diversity within the field. In conclusion, it is essential to promote and support women’s participation in neurosurgery, ensuring they are not discouraged by the demanding nature of the profession.

## Ethical approval

Ethical approval was not applicable for this article.

## Consent

Written informed consent is not applicable for this article.

## Sources of funding

None.

## Conflicts of interest disclosures

None.

## Author contribution

A.A.: conceptualization, validation, visualization, supervision, writing – original draft, and writing – review and editing; I.A.I., P.K., A.A., Y.A., R.K.W., P.S., and S.R.: writing – original draft, and writing – review and editing. All authors approve the final version of the manuscript and are accountable for all aspects of the work.

## Research registration unique identifying number (UIN)

None.

## Guarantor

Ayush Anand.

## Data availability statement

Data sharing is not applicable to this article.

## Provenance and peer review

Not commissioned, externally peer-reviewed.
